# Novel Approaches in Glucose and Lipid Metabolism Disorder Therapy: Targeting the Gut Microbiota–Bile Acid Axis

**DOI:** 10.3390/biology14070802

**Published:** 2025-07-02

**Authors:** Jin Jiang, Huange Zhang, Muhammad Hussain, Abdullah, Fengqin Feng, Rongfa Guan, Hao Zhong

**Affiliations:** 1 College of Food Science and Technology, Zhejiang University of Technology, Hangzhou 310014, China; 2 College of Biosystems Engineering and Food Science, Zhejiang University, Hangzhou 310058, China

**Keywords:** glucose and lipid metabolism, gut microbiota, bile acids, FXR, TGR5

## Abstract

Metabolic dysregulation involving glucose and lipids is closely associated with chronic diseases such as type 2 diabetes mellitus. Emerging evidence highlights the regulatory role of bile acid (BA)–gut microbiota interactions in these metabolic disorders. The gut microbiota orchestrates the biotransformation of primary BAs into bioactive secondary BAs, which function as endocrine signaling molecules by activating the nuclear farnesoid X receptor (FXR) and G protein-coupled membrane receptor (TGR5), forming a communication network essential for metabolic homeostasis. BAs also reciprocally modulate gut microbiota composition. This BA–gut microbiota co-metabolism has emerged as a promising therapeutic target for lipid metabolism disorders. This comprehensive review examines the bidirectional interplay between gut microbiota and BA metabolism, focusing on microbial transformation of BAs, host–microbial co-regulatory pathways and mechanisms of BA metabolism, and the therapeutic implications of modulating the gut microbiota–BA axis in addressing glucose and lipid metabolism disorders. The synthesis of current evidence aims to elucidate the intricate crosstalk between microbial ecology and host metabolism mediated by BA signaling pathways, thereby exploring novel therapeutic intervention strategies.

## Simple Summary

Glucose and lipid metabolism disorders, including diabetes and obesity, are growing global health concerns. Recent advances in histological techniques have revealed how gut microbiota interact with bile acids to form the gut microbiota–bile acid axis—a key regulator of metabolic health. Gut microbes modify bile acids, which influence metabolism by regulating signaling pathways like the farnesoid X receptor and G protein-coupled membrane receptor. This review examines bile acid synthesis, microbial transformation, and their bidirectional crosstalk, with emphasis on how probiotics, polysaccharides, and other interventions can reshape gut microbiota composition and BA profiles to ameliorate glucose and lipid metabolism disorders, thereby uncovering novel therapeutic avenues.

## 1. Introduction

Dysregulation of glucose and lipid metabolism serves as the shared pathological underpinning of metabolic disorders, including obesity, type 2 diabetes mellitus (T2DM), and metabolic dysfunction-associated steatotic liver disease (MASLD). Although its pathogenesis remains incompletely elucidated, accumulating evidence underscores the pivotal role of the gut microbiota in the regulation of glucose and lipid metabolism [1]. The structure and metabolic functions of the intestinal microbiota play a crucial role in modulating the glucose homeostasis and lipid metabolic balance. On one hand, microbial dysbiosis may facilitate the translocation of metabolic endotoxins (e.g., lipopolysaccharide) into systemic circulation, thereby inducing chronic, persistent, low-level inflammation and impaired insulin sensitivity. On the other hand, gut microbiota enzymatically modifies bile acids (BAs) through biotransformation processes (e.g., deconjugation, 7α-dehydroxylation, oxidation, epimerization, and reconjugation). These microbially transformed BAs not only act as critical mediators of fat digestion and absorption but also function as pleiotropic bioactive mediators, orchestrating host glucose and lipid metabolism, inflammatory responses, and energy homeostasis [2].

Recent studies highlight the critical role of the gut microbiota–BA axis in host glycolipid metabolism. Sun et al. revealed that metformin’s antidiabetic action is primarily mediated through intestinal flora regulation and BA metabolic pathways. Co-metabolic networks between Bas, gut microbiota, and the host are considered therapeutic targets for various metabolic diseases [3]. Specifically, gut microbiota convert primary BAs (e.g., cholic acid (CA) and chenodeoxycholic acid (CDCA)) into secondary BAs (e.g., DCA and LCA) via bile salt hydrolase (BSH) and other enzymes, thereby altering their bioactivity, while these secondary BAs activate key receptors such as the farnesoid X receptor (FXR) and Takeda G-protein receptor 5 (TGR5) to modulate gluconeogenesis, lipid oxidation, and energy metabolism, ultimately influencing systemic metabolic homeostasis [4]. Understanding these mechanisms has facilitated the design of innovative treatments targeting this axis, including probiotics, dietary interventions, and FXR/TGR5-targeting drugs, which offer innovative approaches for preventing and treating metabolic disorders. Further in-depth research on the gut microbiota–BA–host metabolic network will not only elucidate the molecular basis of metabolic dysregulation but also establish a robust theoretical foundation for developing next-generation metabolic therapies.

This systematic review endeavors to elucidate intricate interactions between BAs and gut microbiota, unravel the underlying mechanisms through which gut microbiota modulate BA metabolism, and comprehensively synthesize the regulatory mechanisms of the gut microbiota–BA axis in maintaining glucose and lipid metabolism homeostasis. Additionally, it offers a comprehensive review of innovative therapeutic approaches targeting the gut microbiota–BA axis (e.g., diets, probiotics and prebiotics, fecal microbiota transplantation (FMT), and secondary BAs), providing novel perspectives for the clinical management of metabolic disorders characterized by dysregulated glucose and lipid metabolism.

## 2. Glycolipid Metabolism Disorders and Metabolic Diseases

Glycolipid metabolism represents a fundamental physiological process governing the biochemical conversion of carbohydrates and lipids across major metabolic organs including the liver, skeletal muscle, adipose tissue, and pancreatic islets, playing a pivotal role in maintaining systemic energy homeostasis [5]. This complex metabolic network comprises two interdependent pathways: carbohydrate metabolism involving the digestion of dietary carbohydrates into glucose for immediate energy use or hepatic glycogen storage [6], and lipid metabolism encompassing the absorption and storage of dietary triglycerides in adipocytes [7]. Disruptions in glucose metabolism frequently precipitate hyperglycemia and insulin resistance, subsequently triggering excessive lipolysis, abnormal triglyceride accumulation, elevated low-density lipoprotein cholesterol (LDL-C) levels, and reduced high-density lipoprotein cholesterol (HDL-C) concentrations [8]. Conversely, impaired lipid metabolism can compromise glucose oxidation pathways and induce pancreatic β-cell dysfunction through lipotoxic mechanisms, potentially leading to β-cell programmed cell death and worsening glucose dysregulation. The endocrine pancreas orchestrates these metabolic processes through the counterregulatory actions of insulin and glucagon. Insulin promotes glycogenesis, facilitates glucose transport into cells, suppresses glucose production by the liver, and inhibits lipolysis [6], while glucagon stimulates gluconeogenesis and glycogenolysis to elevate blood glucose levels. Additionally, Glucagon-like peptide-1 (GLP-1) exerts glucose-dependent effects by augmenting insulin secretion, inhibiting glucagon release, and supporting β-cell regeneration, thereby ameliorating hyperglycemia in T2DM patients [9].

Numerous factors contribute to glycolipid metabolic disorders through complex interactions between genetic predisposition and environmental influences. Genetic determinants significantly influence susceptibility to diabetes, hypertension, and dyslipidemia, while lifestyle factors including physical inactivity and consumption of energy-dense diets rich in refined sugars and saturated fats exacerbate metabolic dysfunction. The liver serves as the central metabolic hub for both glucose and lipid synthesis, with excessive nutrient intake leading to elevated circulating levels of these metabolites. Insulin resistance, characterized by diminished cellular responsiveness to insulin, impairs glucose uptake while permitting unrestrained lipolysis [10], thereby promoting hyperglycemia and dyslipidemia, which underlie T2DM pathogenesis. Various hormones including catecholamines, glucagon, and thyroid hormones, critically modulate metabolic balance. Chronic low-grade inflammation associated with obesity and other pathological conditions further aggravates insulin resistance and metabolic derangements [11]. Alterations in gut microbial composition affect nutrient fermentation and metabolite production, while certain pharmacological agents such as antipsychotics and antihypertensive medications may adversely impact glycolipid metabolism [12].

Persistent glycolipid metabolic disturbances give rise to multiple pathological conditions through interconnected mechanisms. T2DM develops as a consequence of progressive β-cell failure in the context of chronic insulin resistance, manifesting as sustained hyperglycemia [13]. The reciprocal relationship between insulin resistance and obesity creates a vicious cycle that perpetuates metabolic dysfunction. Compelling evidence demonstrates that glucose and lipid metabolism disorders contribute to hypertension pathogenesis via insulin resistance-mediated vascular dysfunction and abnormal fluid homeostasis [14]. These metabolic abnormalities also promote atherogenic dyslipidemia featuring elevated cholesterol and triglyceride levels, substantially increasing cardiovascular disease risk [15]. The concurrent presence of hypercholesterolemia, hypertension, and diabetes markedly accelerates atherosclerosis development and raises the likelihood of major adverse cardiac events. Furthermore, disrupted glucolipid homeostasis leads to pathological lipid accumulation in hepatocytes, predisposing to MASLD and its progressive sequelae [16]. Emerging research suggests that chronic glycolipid metabolic disorders may also contribute to polycystic ovary syndrome through insulin resistance-mediated hormonal imbalances and have been implicated in the pathogenesis of certain neurodegenerative disorders and malignancies through mechanisms involving chronic inflammation and oxidative stress. The broad spectrum of metabolic diseases arising from glycolipid dysregulation underscores the critical importance of maintaining metabolic homeostasis for overall health.

## 3. Gut Microbiota–BA Axis

The gut microbiota actively transforms bile acids (BAs), influencing both their molecular composition and pool size. In turn, BA modifications can reciprocally reshape microbial community structure and population dynamics.

### 3.1. Gut Microbiota Mediates BA Metabolism

**Figure 1:**
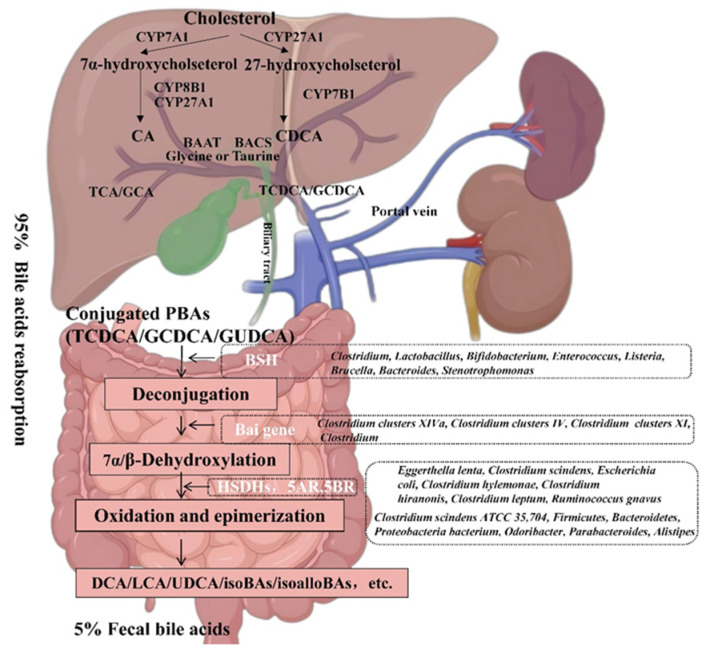
Enterohepatic circulation of bile acids in humans.

BA biosynthesis occurs in the liver through two distinct pathways, with the classical route being dominant in humans and responsible for more than three-quarters of total production, while the alternative pathway contributes the remainder primarily through CYP27A1-initiated oxidation of cholesterol. Primary bile acids (PBAs), such as CA and CDCA, are synthesized from cholesterol through enzymatic reactions mediated by cytochrome P450 (CYP) enzymes in microsomes, notably cholesterol 7α-hydroxylase (CYP7A1) and mitochondrial sterol 27-hydroxylase (CYP27A1). Following their synthesis, these primary BAs undergo activation by BA-CoA synthase (BACS) to form CoA derivatives, which then enter a conjugation process facilitated by BA-CoA-amino acid N-acyltransferase (BAAT). This enzymatic process predominantly yields glycine-conjugated species in humans, while taurine-conjugated forms are more prevalent in rodents [17]. The conjugated BAs are then exported from hepatocytes into bile canaliculi through the bile salt export pump (BSEP). After traversing the bile duct network and undergoing concentration in the gallbladder, these BAs are ultimately released into the duodenal lumen during digestion [18]. The enterohepatic circulation efficiently reclaims approximately 95% of intestinal BAs through active ileal absorption, with the residual 5% being eliminated in feces. This recycling process repeats 4–12 times daily, ensuring optimal bile acid economy [19]. (Figure 1) Intestinal microbiota significantly reshape the BA profile through multiple biochemical transformations: (1) BSHs cleave conjugated BAs to generate free BAs; (2) The *bai* (bile acid inducible) gene cluster, which encodes enzymes responsible for 7α/7β-dehydroxylation, mediates the conversion of primary BAs to secondary BAs (lithocholic acid (LCA) and deoxycholic acid (DCA)) in the colon; (3) Microbial hydroxysteroid dehydrogenases (HSDHs) catalyze oxidation, epimerization (yielding β-hydroxy isocholic acids), and position-specific hydroxylation (e.g., at C6 to form muricholic acids). The latter includes α-muricholic acid (αMCA; 3α,6β,7α-trihydroxy-5β-cholan-24-oic acid) and β-muricholic acid (βMCA; 3α,6β,7β-trihydroxy-5β-cholan-24-oic acid), which are particularly abundant in murine systems but also detectable in human infants [20]. Furthermore, BAs are characterized by a distinctive cyclopentanophenanthrene steroid core, wherein the spatial arrangement of functional groups exhibits specific orientation: the hydrophilic α-hydroxyl groups extend above the planar nucleus, whereas the opposing surface maintains its hydrophobic nature, thereby endowing these molecules with amphiphilic properties. This unique architecture enables BAs to effectively reduce interfacial tension at lipid–water boundaries [21]. The majority of hepatic BAs exist as taurine or glycine conjugates, modifications that serve dual purposes: (1) enhancing aqueous solubility by lowering pKa, and (2) restricting passive membrane permeability while decreasing critical micellar concentration. These physicochemical characteristics are essential for their physiological roles in dietary fat emulsification and promoting the uptake of fat-soluble vitamins [22]. The enterohepatic recirculation mechanism ensures the optimal recycling and metabolic economy of these biologically active molecules.

### 3.2. BAs Remodel the Gut Microbiota Structure

BAs modulate the intestinal microbial community by promoting the growth of bacterial species capable of BA metabolism while suppressing bile-sensitive bacteria. In instances of biliary obstruction, the compromised bile flow induces bacterial overgrowth within the small intestine, a pathological condition that can be ameliorated through BA supplementation [23]. CA supplementation in rats significantly altered the composition of the gut microbiota at the phylum level, marked by an increase in Firmicutes and a decrease in Bacteroidota [24]. Ursodeoxycholic acid (UDCA) transiently elevated hepatic triglyceride (TG) levels [25], whereas tauroursodeoxycholic acid (TUDCA) demonstrated anti-inflammatory properties and enhanced insulin sensitivity [26]. Glycoursodeoxycholic acid (GUDCA) reduced the abundance of *Pseudomonas corrugata* and promoted *Bacteroides vulgatus* growth. Notably, *Bacteroides vulgatus* positively correlates with taurolithocholic acid (TLCA) and non 12α-hydroxy BAs, while *Pseudomonas corrugata* associates with elevated 12α-hydroxy/non 12α-hydroxy BA ratios and PBA/SBA levels [27]. Additionally, BAs regulate gut microbiota through multiple nuclear and membrane receptors, including FXR, TGR5, vitamin D receptor, and pregnane X receptor. For example, the FXR agonist obeticholic acid (OCA) suppresses endogenous BA synthesis while promoting gram-positive bacteria (e.g., *Streptococcus salivarius subsp. thermophilus*, *Lacticaseibacillus paracasei*, *Bifidobacterium breve*, and *Lactococcus lactis* [28]). This receptor also mediates microbiota shifts in response to fluctuating BA levels in the small intestine. Centenarians (*n*  =  160; mean age, 107 years) demonstrated a distinct microbial profile characterized by dominance of *Bacteroides* and *Alistipes*, contrasting with suppressed *Streptococcus* levels in younger cohorts [29]. They also exhibited a distinct BA profile—elevated alloLCA, isoLCA, 3-oxoalloLCA 3-oxoLCA, and isoalloLCA. Notably, isoalloLCA directly inhibits C. difficile 630 and replicates centenarian-like microbiota changes in vitro: suppressing gram-positive populations (e.g., *Faecalibacterium prausnitzii*, *Bifidobacterium*, and *Streptococcus*) while enhancing gram-negative (e.g., *Bacteroides* and *Alistipes*) populations. These findings highlight isoalloLCA’s potential role in pathogen resistance and microbiota homeostasis [30].

BAs also function as critical antimicrobial components of intestinal innate immunity. Their bactericidal mechanism primarily involves cell membrane disruption, leading to intracellular content leakage [31]. Notably, unconjugated BAs (e.g., CDCA and DCA) exhibit stronger antimicrobial activity than conjugated forms (e.g., GCA and TCA) due to their enhanced membrane permeability at pH7. This property makes unconjugated BAs particularly effective against *Staphylococcus aureus*. Structural modifications further enhance BA antimicrobial potential: six CA derivatives obtained from *Bacillus amyloliquefaciens* UWI-W23 cultures show potent activity against microbes [32]. Furthermore, LCA and its derivatives demonstrate broad-spectrum effects against *Escherichia coli*, *Staphylococcus aureus subsp. anaerobius*, *Bacillus cytotoxicus*, and *Pseudomonas aeruginosa* [33]. These findings highlight BAs’ promise as scaffolds for novel antibiotic development against resistant pathogens. Overall, specific BAs contribute to metabolic balance, and their supplementation may influence gut microbial composition [29].

## 4. The Mechanisms of Bile Acid Metabolism by Gut Microbiota

**Figure 2:**
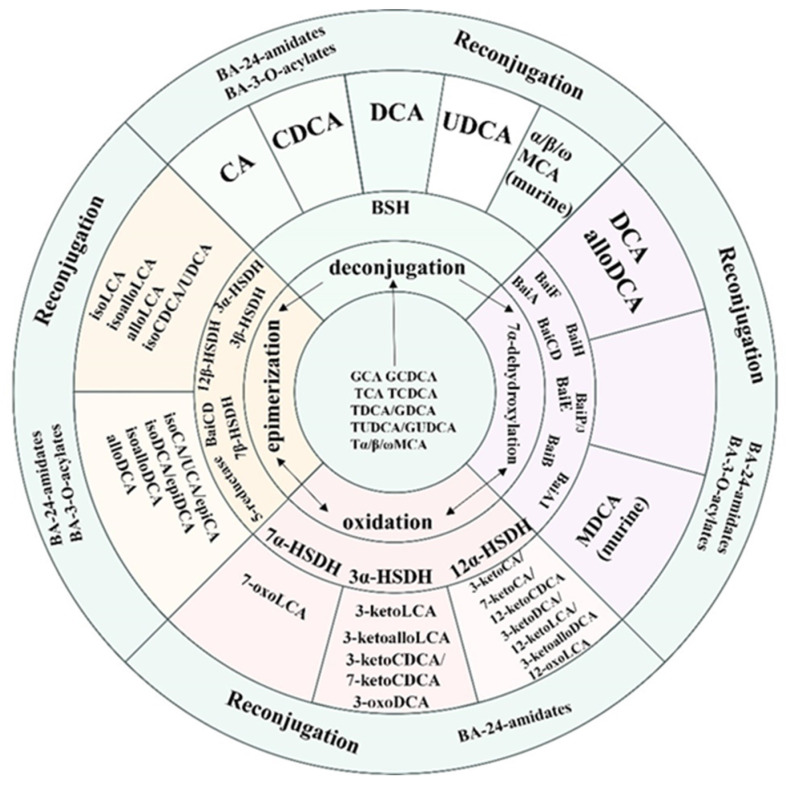
Gut microbial-derived modifications of bile acids in the human intestinal tract.

**Figure 3:**
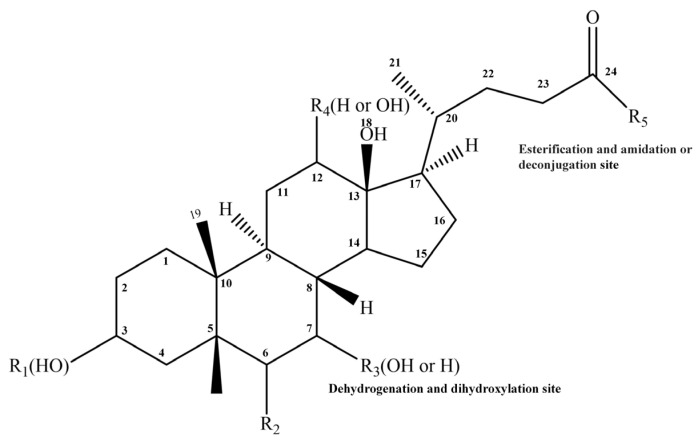
General bile acid structure.

Gut microbiota enzymatically transform primary BAs in the colon via multi-step modifications including deconjugation, structural rearrangement (7α/β-dehydroxylation and oxidation/epimerization), and reconjugation processes (Figure 2). These microbial modifications not only enhance the structural diversity of BAs (as illustrated by their general structure in Figure 3) but also increase the overall hydrophobicity of the BA pool, thereby influencing their physiological functions.

### 4.1. Deconjugation

Primary BAs (such as CA and CDCA) originate from hepatic synthesis, where they are typically converted into glycine- or taurine-conjugated forms. Upon entering the intestine, gut microbiota (particularly *Listeria*, *Enterococcus*, *Clostridium*, *Lactobacillus*, *Bifidobacterium*, *Brucella*, *Bacteroides*, and *Stenotrophomonas*) express BSH, which catalyzes the deconjugation reaction through the cleavage of amide bonds, consequently liberating free BAs (e.g., CA and CDCA) accompanied by glycine or taurine [34]. This process not only enhances the diversity of the BA pool but also significantly increases its overall hydrophobicity, subsequently influencing host lipid absorption, glucose metabolism, and energy homeostasis. Different bacterial strains exhibit substrate selectivity in their BSH enzymes. For instance, the three BSH isoforms in *Lactobacillus johnsonii* (*L. johnsonii*) PF01 preferentially deconjugate glycine-conjugated BAs, while the two isoforms (BSH1 and BSH2) in *Lactobacillus salivarius* LMG14476 display distinct substrate preferences. Notably, the BSH variants present in *L. johnsonii* PF01 demonstrate significantly higher enzymatic activity toward glycine-conjugated bile salts compared to their taurine-conjugated counterparts [35]. Notably, the BSH-t3 enzyme exhibits particularly pronounced activity among *Lactobacillus* species within the human gut microbiota, a phenomenon that presumably reflects their pivotal role in BA metabolism [36].

### 4.2. 7α/7β-Dehydroxylation

The gut microbiota performs profound structural remodeling of BA molecules through highly specific 7α/7β-dehydroxylation, a complex biotransformation process coordinated by multiple tightly regulated enzymatic systems. In the anaerobic intestinal environment, dominant bacterial groups such as Clostridium and Eubacterium initiate a multi-step catalytic cascade via the BA-inducible (*bai*) operon. This process begins with the uptake of primary BAs into bacterial cells through the *BaiG* transporter, followed by *baiB*-mediated CoA ligation that converts CA or CDCA into activated cholyl-CoA thioesters [37]. This activated form subsequently undergoes baiA2-catalyzed C3 oxidation and baiCD-mediated 4,5-dehydrogenation, forming the critical 3-oxo-4,5-dehydrocholyl-CoA intermediate, which is ultimately converted by *baiE* through specific 7α-dehydroxylation into 3-oxo-4,5-6,7-didehydro-deoxycholyl-CoA [38]. Notably, different bacterial strains exhibit significant metabolic heterogeneity—for instance, *Faecalicatena contorta* S122 maintains active *bai* operon expression despite its low intestinal abundance [39], while *Eggerthella lenta*, though harboring relevant genes, lacks actual transformation capability [40]. Beyond the classical 7α-dehydroxylation pathway, certain specialized strains can catalyze 7β-dehydroxylation to generate UDCA, a compound with unique pharmacological activity, or induce 6β-hydroxylation in mouse intestines to produce rodent-specific BA derivatives. These microbially modified products exert profound regulatory effects on host glucose and lipid metabolism homeostasis through the activation of nuclear receptor FXR and membrane receptor TGR5, with DCA and LCA exhibiting particularly prominent dual effects: on one hand, DCA may contribute to colorectal carcinogenesis through DNA damage induction and cell proliferation promotion, while on the other hand, UDCA demonstrates remarkable hepatoprotective effects and is widely used in clinical treatment for cholestatic liver diseases. Recent studies have not only successfully reconstituted the complete 7α-dehydroxylation enzyme system in vitro, revealing the evolutionary conservation of *bai* enzymes among Firmicutes, but also identified significant structural differences between 7α- and 7β-dehydroxylases. These scientific breakthroughs establish a fundamental theoretical framework for the development of targeted intervention strategies aimed at modulating gut BA metabolism. This sophisticated microbial chemical transformation network not only significantly enhances the structural diversity of BA molecules but also serves as a pivotal component in host–microbial co-metabolic regulation. A comprehensive elucidation of this network will provide novel therapeutic approaches for addressing metabolic disorders.

### 4.3. Oxidation and Epimerization

The gut microbiota orchestrates profound structural remodeling of BA molecules through intricate oxidation and epimerization processes, a sophisticated biotransformation primarily mediated by highly specific hydroxysteroid dehydrogenases (HSDHs) [41]. Within the intestinal ecosystem, diverse microbial species including *Hungatella hathewayi* [42], *Eubacterium* spp. [43], *Hungatella hiranonis* [42], *Eggerthia lenta* [44], *Escherichia coli* [45], and *Bacteroides fragilis* [46] catalyze reversible oxidation–reduction reactions at various hydroxyl positions (3α-, 3β-, 7α-, or 12α-) of BAs through their encoded HSDHs. Notably, the *Eggerthella* sp. CAG:29815 strain harbors NAD(P)H-dependent 3α-, 3β-, and 12α-HSDH gene clusters, while the 12α-HSDH coding gene is present in *Eggerthella* sp. CAG:298, *Hungatella hathewayi*, *Hungatella hylemonae*, and *Peptacetobacter hiranonis* [42]. These enzymatic modifications not only alter BAs’ physicochemical properties but also generate more hydrophilic and less toxic epimeric BAs (isoBAs) [47]. A prime example is the microbial conversion of hydrophobic CDCA to its 7β-epimer UDCA, a structural transformation that significantly enhances bacterial competitive fitness in the gut environment [47,48]. Remarkably, these epimeric BAs also play crucial roles in modulating gut microbiota composition and host metabolism. For instance, *Eggerthella lenta* produces 3-oxodeoxycholic acid (3-oxoDCA) via specific 3α-HSDH activity, while *Ruminococcus gnavus* generates epideoxycholic acid (isoDCA) through 3β-HSDH catalysis, with the latter shown to promote *Mycobacterium* spp. growth [44]. Cutting-edge research has further uncovered the pivotal role of two bacterial steroid dehydrogenases—5α-reductase (3-oxo-5α-steroid 4-dehydrogenase, 5AR) and 5β-reductase (3-oxo-5β-steroid 4-dehydrogenase, 5BR)—in the biosynthesis of homologous (5α and 5β) BAs. Centenarian populations (average age 107 years) exhibit significantly elevated levels of 3-oxolithocholic acid (3-oxoLCA), isolithocholic acid (isoLCA), 3-oxoallolithocholic acid (3-oxoalloLCA), allolithocholic acid (alloLCA), and isoallolithocholic acid (isoalloLCA), attributable to their unique gut microbiota composition. Microbiome analysis of these long-lived individuals revealed numerous bacterial strains with potent 3α-HSDH, 3β-HSDH, 5AR, and/or 5BR activities [29]. Current evidence suggests that 3-oxoalloLCA likely originates from 3-oxo-△4-LCA via 5AR homologs, with subsequent conversion to alloLCA or isoalloLCA mediated by 3α-HSDH or 3β-HSDH respectively—a transformation mechanism strikingly similar to the previously described conversion of 3-oxoDCA to DCA or isoDCA [30,49,50]. These discoveries not only expand our understanding of the gut microbiota–host co-metabolic network but also provide novel theoretical foundations for developing intervention strategies targeting BA metabolism regulation.

### 4.4. Reconjugation

The gut microbiota orchestrates an intricate reconjugation network that reshapes BA structure and function through two principal biochemical transformations: amidation at the C-24 position with various amino acids or polyamines to generate BA-24-amidates, and esterification at the C-3 hydroxyl group with fatty acids or organic acids yielding BA-3-O-acylates. This metabolic reprogramming involves a consortium of intestinal bacteria spanning multiple genera, with *Bacteroides*, *Lactobacillus*, and *Bifidobacterium* demonstrating particularly robust conjugation capabilities alongside *Enterocloster*, *Ruminococcus*, and select *Clostridium* species [51,52]. While BSH has traditionally been recognized for its deconjugating activity, emerging evidence reveals its unexpected participation in reamidation processes, with certain bacterial strains like *Clostridium scindens* ATCC 35704 exhibiting conjugation proficiency even in the absence of canonical *bsh* genes [51]. The resulting reconjugated BAs display remarkable functional diversity, with phenylalano and tyroso conjugates emerging as potent FXR agonists capable of modulating hepatic BA synthesis [53], while CDCA derivatives demonstrate pleiotropic receptor activation spanning FXR, PXR, and AHR pathways [52]. Conversely, hyocholic and hyodeoxycholic acid conjugates function as FXR antagonists that stimulate intestinal GLP-1 secretion.

Comprehensive metabolomic profiling has unveiled an extensive array of 3-O-acylated derivatives encompassing both primary (CA and CDCA) and secondary (DCA and LCA) BAs conjugated with short-chain (C1–C5) and long-chain (C16–C18) fatty acids [2,53], with *Bacteroides* species serving as primary drivers of this esterification process [54]. Notably, *Christensenella* strains exhibit exceptional substrate promiscuity in SCFA conjugation reactions [55], while *Bacteroides uniformis* employs a specialized β-lactamase (BAS-suc) for succinyl-CA formation [1]. These microbial modifications carry significant metabolic implications, as evidenced by the selective depletion of 3-acyl-CA derivatives in T2DM [1] and the therapeutic potential demonstrated by 3-succinylCA in ameliorating MASH pathology through *Akkermansia muciniphila* enrichment [56]. The expanding repertoire of microbially reconjugated BAs represents a promising yet underexplored therapeutic frontier for metabolic disorders, though complete mechanistic understanding of their biosynthesis and physiological impacts awaits further investigation.

## 5. The Mechanism of Interaction Between Bile Acids and Gut Microbiota in Improving Glucose and Lipid Metabolism

As a pivotal regulatory hub in host–microbial co-metabolism, the gut microbiota orchestrates the synthesis, metabolism, and reabsorption of BAs, consequently modulating BA composition, pool size, and their physiological functionalities. BAs perform essential functions not only in facilitating lipid digestion and absorption but also in regulating hepatic metabolism, insulin sensitivity, and energy homeostasis through their interactions with nuclear receptors (e.g., FXR). Accordingly, accumulating evidence indicates that targeting the BA–gut microbiota axis constitutes an innovative therapeutic approach for addressing glucose and lipid metabolism disorders (Table 1).

Studies indicate that disruption of BA homeostasis or related signaling pathways can lead to cholestasis, hepatobiliary injury, and metabolic dysregulation [84]. BA homeostasis is predominantly sustained through negative feedback mechanisms and spatiotemporally regulated enterohepatic circulation. The regulation of BA synthesis, primarily governed by the activation of FXR in both hepatic and intestinal tissues, detects BA concentrations and modulates the transcription of genes associated with BA synthesis, conversion, transport, and signaling pathways [85]. Upon reabsorption in the ileum, BAs activate intestinal FXR, thereby stimulating the hepatic synthesis of the small heterodimer partner (SHP) protein and FGF19. FGF19, along with reabsorbed BAs, enters the portal circulation and suppresses hepatic BA synthesis, thereby maintaining BA pool homeostasis [86].

**Figure 4:**
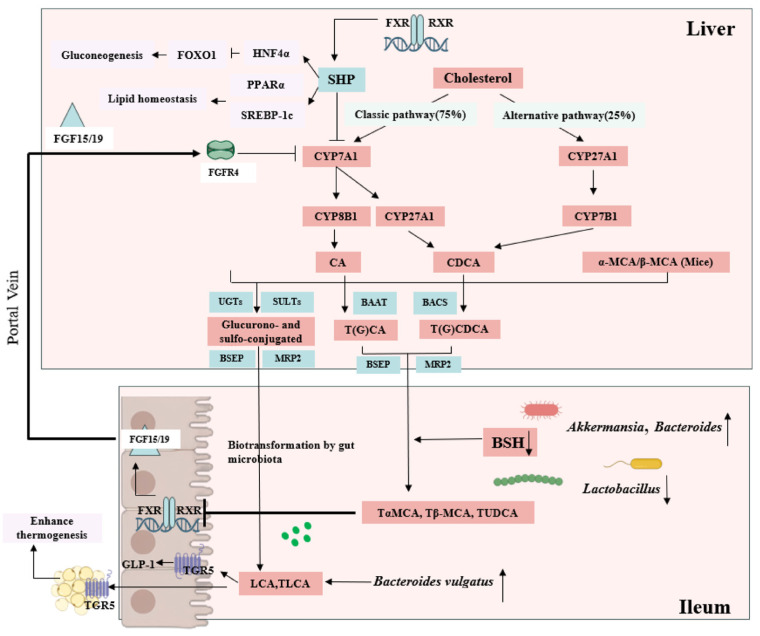
Mechanisms of targeting the gut microbiota–BA axis to regulate glucose and lipid metabolism.

Emerging research has elucidated the tissue-specific duality of FXR signaling in metabolic regulation, revealing distinct therapeutic implications for hepatic versus intestinal FXR modulation. While intestinal FXR inhibition has demonstrated efficacy against obesity, diabetes, and MASLD through mechanisms such as suppressing hepatic gluconeogenesis via SHP-independent enterohepatic signaling [87,88] and reducing hepatic lipid accumulation through suppression of SHP and FGF15/FGF19 expression, contrasting evidence highlights the metabolic benefits of hepatic FXR activation. Liver-specific FXR stimulation exerts glucose-lowering effects through the FXR-SHP-HNF4α/Foxo1 cascade [89] while simultaneously improving lipid homeostasis via coordinated downregulation of SREBP-1c and upregulation of PPARα pathways [90] (Figure 4). This apparent paradox stems from FXR’s capacity to differentially regulate tissue-specific gene networks, where the same receptor can mediate opposing metabolic outcomes depending on its anatomical location and downstream signaling effectors. The compartmentalized nature of FXR signaling underscores the importance of developing tissue-targeted FXR modulators for metabolic disorders, as systemic FXR activation or inhibition may produce conflicting physiological effects due to the receptor’s distinct regulatory roles in different organ systems. This section will systematically examine how emerging therapeutic approaches modulate glucose and lipid homeostasis via the gut microbiota–bile acid–FXR/TGR5 signaling axis.

### 5.1. Novel Approaches in Improving Glucose and Lipid Metabolism Through the Gut Microbiota–Bile Acid–FXR/TGR5 Axis

Gut microbiota-derived BA modifications (3-O-acylation and MCY conjugation) and microbial transformation products (e.g., TLCA) orchestrate glucose/lipid homeostasis by antagonizing intestinal FXR and activating TGR5, respectively, offering novel therapeutic avenues for metabolic disorders. Recent studies demonstrate that gut commensal *Christensenella minuta* biosynthesizes unique 3-O-acylated secondary BAs capable of selectively inhibiting intestinal FXR activity and enhancing glucose and lipid homeostasis. The discovery of these microbially generated FXR inhibitors provides new insights into host–microbe metabolic crosstalk and suggests potential therapeutic strategies for metabolic disorders via targeted intestinal FXR suppression [1]. Won et al. discovered a group of BA–methylcysteamine (BA–MCY) conjugates that are highly prevalent in the intestinal tract. BA–MCYs serve as strong FXR antagonists, enhancing the expression of BA biosynthesis-related genes in vivo. In hypercholesterolemic mice, administration of stable isotope-labeled BA–MCY compounds significantly enhanced BA production through an FXR-dependent mechanism and reduced hepatic lipid accumulation [82]. GUDCA intervention upregulated levels of UDCA and its taurine/glycine conjugates (TUDCA and GUDCA), as well as LCA and its derivatives (isoLCA and TLCA), alongside an elevated abundance of *Bacteroides vulgatus*. TLCA, as a TGR5 agonist, upregulates UCP-1 expression, thereby enhancing thermogenesis in WAT. Additionally, it stimulates intestinal GLP-1 secretion and promotes thermogenic activity in BAT and skeletal muscle. Through these mechanisms, GUDCA may restore metabolic balance in glucose and energy homeostasis by modulating the TLCA-*B*. *vulgatus*–TGR5 axis [27,91]. GUDCA supplementation attenuates atherosclerosis in high cholesterol-fed ApoE^−/−^ mice by suppressing intestinal FXR signaling and lowering circulating ceramide levels [83]. Additionally, the polysaccharide from *Lyophyllum decastes* exerts anti-obesity effects by modulating gut microbiota composition, particularly enriching *Bacteroides intestinalis* and *Lacticaseibacillus johnsonii*, which promotes the production of secondary BAs, including hyodeoxycholic acid (HDCA), DCA, and LCA. These BAs act as potent TGR5 agonists, inducing browning of sWAT and stimulating thermogenic activity in BAT, thereby enhancing energy expenditure. Mechanistically, this process involves the upregulation of key thermogenic markers such as uncoupling protein 1 (Ucp1), peroxisome proliferator-activated receptor γ (PPARγ), coactivator-1α (Pgc1α), and transcription factor PR domain containing 16 (Prdm16), all of which are involved in energy metabolism. The activation of the TGR5 signaling pathway by elevated secondary BAs drives BAT-mediated energy expenditure, representing a central mechanism through which the polysaccharide alleviates obesity [59].

Emerging evidence reveals that modulation of the gut microbiota–BA axis through suppression of BSH activity, alteration of BA composition (e.g., elevated TβMCA/TUDCA), and inhibition of intestinal FXR signaling effectively enhances hepatic BA synthesis (via CYP7A1) and improves glucose/lipid homeostasis, offering novel therapeutic strategies for metabolic disorders. Antibiotic treatment in animals induced CYP7B1 upregulation, shifting BA composition toward more hydrophilic species, notably tauro-β-muricholic acid (TβMCA). This shift suppressed intestinal FXR signaling and ameliorated high-fat diet (HFD)-induced metabolic dysfunction, including glucose intolerance, hepatic steatosis, and systemic inflammation. Mechanistically, these improvements were associated with reduced hepatic de novo lipogenesis and elevated thermogenic activation in subcutaneous white adipose tissue (sWAT) [92]. Similarly, Capsaicin (CAP) modulated gut microbiota by suppressing *Lactobacillus*-mediated BSH activity to elevate TβMCA levels. This process inhibits enterohepatic FXR-FGF15 signaling, subsequently expanding the BA pool through upregulated CYP7A1 expression and enhanced hepatic BA synthesis [62]. Tomato pectin (TP) treatment significantly altered gut microbiota composition and BA metabolism in HFD-fed mice, demonstrating therapeutic potential for hepatic steatosis. The intervention reduced *Lactobacillus* and *Romboutsia* abundance while promoting growth of Muribaculaceae, *Blautia*, *Bacteroides*, *Akkermansia*, Rikenellaceae_RC9_gut_group, *Alloprevotella*, *Parabacteroides*, and Peptococcaceae. Concurrently, TP administration markedly decreased BSH activity in both stool and ileum samples, accompanied by elevated fecal levels of tauro-conjugated BAs (TαMCA, TβMCA, TωMCA, TCDCA, TDCA, and TUDCA) and increased 12KLCA, βMCA, ωMCA, CA, HCA, and UCDA content. In contrast, the concentrations of CDCA, LCA, and DCA were substantially reduced. These changes collectively contributed to the amelioration of HFD-induced hepatic steatosis through gut microbiota restructuring, BSH activity suppression, and FXR signaling modulation [61].

Traditional Chinese Medicine (TCM) may also improve glucose and lipid metabolism by modulating the gut microbiota–BA–FXR interaction network. Gyejibongnyeong-hwan (GBH) treatment modulated gut microbiota composition by reducing *Eisenbergiella massiliensis*, *Roseburia faecis*, and *Pseudoflavonifractor capillosus* bacteria positively associated with hydrophobic BAs (CDCA, TCDCA, LCA, and TLCA). The subsequent decrease in CDCA and LCA (potent FXR ligands) inhibited intestinal FXR-FGF15 signaling, while upregulating hepatic genes involved in cholesterol metabolism (liver X receptor alpha LXRα and ATP-binding cassette subfamily G member 8 ABCG8) and BA synthesis (CYP7A1). Together, these effects improved Western diet-induced dyslipidemia via the gut microbiota–BA axis [69]. Simiao Wan (SMW) is a classic Chinese medicine prescription first recorded in the *Cheng Fang Bian Du* of the Qing Dynasty. Wang et al. demonstrated that Simiao Wan (SMW) ameliorated HFD-induced hyperlipidemia by reducing both intrahepatic and white adipose tissue (iWAT) fat accumulation. The therapeutic effects were mediated through multiple pathways: (1) suppression of BSH-producing bacteria and consequent BSH activity inhibition, leading to elevated conjugated BA levels (particularly TβMCA and TUDCA); and (2) modulation of FXR signaling, where ileal FXR-FGF15 pathway inhibition promoted BA efflux and subsequently activated the hepatic CYP7A1/FXR/SHP axis, ultimately reducing cholesterol levels and improving lipid profiles [78]. Conversely, other research demonstrates that lipid metabolism improvement correlates with increased BSH-producing bacteria (e.g., *Bifidobacterium*) and elevated unconjugated BAs, as observed with the Zhi-Kang-Yin formula (ZKY). This alternative pathway involves BSH-mediated BA deconjugation, which activates fatty acid degradation pathways and ultimately enhances host glucose and lipid metabolism in preclinical models [76].

Several studies have demonstrated that hepatic FXR activation can also improve glucose and lipid metabolism. Salidroside may serve as a potential therapeutic agent for MASLD by activating FXR. Specifically, it decreases the relative abundance of *Alloprevotella* spp. and *Lactobacillus* while promoting the growth of *Ruminiclostridium* spp. and Lachnospiraceae, thereby restoring microbial balance. Additionally, salidroside lowers TaMCA and TβMCA levels while elevating βCDCA concentrations in the colon, further enhancing FXR activation [70]. Buckwheat dietary fiber (BDF) treatment significantly exhibited significant alterations in gut microbiota composition and BA metabolism in db/db mice, ultimately improving glucose metabolism via the FXR/TGR5 pathway. The abundance of *Akkermansia*, *Bacteroides*, *Lachnoclostridium*, *Coriobacteriaceae_UCG-002*, and *Parabacteroides* increased, while *Erysipelatoclostridium*, *Escherichia-Shigella*, *Lactobacillus*, and *Enterococcus* decreased. These microbial shifts were associated with elevated levels of non-12α-hydroxylated BAs (e.g., ω-MCA, β-MCA, 12-KLCA, LCA-3S, 7,12-DKLCA, and 12-KCDCA) and reduced 12α-hydroxylated BAs (e.g., DCA). Notably, CDCA derivatives (e.g., 12-KCDCA) and LCA derivatives (e.g., 12-KLCA, 7,12-DKLCA) were significantly elevated in the BDF treatment group, alongside upregulated FXR and TGR5 expression. Metabolically, ω-MCA, β-MCA, 12-KLCA, LCA-3S, and 7,12-DKLCA exhibited negative correlations with glycemic markers (FBG, AUC, HbA1c, HOMA-IR, etc.), suggesting their role in glucose regulation. These comprehensive findings provide evidence that BDF alleviates T2DM by modulating the gut microbiota–BA–TGR5/FXR axis, promoting non-12-OH BA biosynthesis, while suppressing 12-OH BAs, thereby activating hepatic FXR/TGR5 signaling [93].

### 5.2. Novel Approaches in Improving Glucose and Lipid Metabolism Through Modulating the Gut Microbiota and Altering the Serum Bile Acid Profile

Emerging evidence highlights modulating the gut microbiota and altering the serum bile acid profile as key regulators of host glucose and lipid homeostasis. QiDiTangShen granules (QDTS), a traditional Chinese herbal medicine, have been used in clinical practice for treating diabetic kidney disease for several years. QiDiTangShen (QDTS) granules ameliorate diabetic nephropathy (DN) by modulating gut microbiota composition and BA metabolism in a diabetic mouse model. Treatment with QDTS reduced the abundance of Muribaculaceae (formerly known as Lachnospiraceae_NK4A136_group), *Lactobacillus*, and *Bacteroides* while increasing *Alloprevotella* levels. Concurrently, QDTS significantly lowered β-MCA, TCA, TβMCA, and DCA levels, which are elevated in T2DM mice. Correlation analysis revealed that renal injury markers (KIM-1, ΔUAE, and KIM-1/Cre ratio) exhibited positive correlations with TβMCA, suggesting a link between BA dysregulation and DN progression. These findings highlight QDTS as a potential therapeutic strategy for DN through gut microbiota and BA homeostasis restoration [77].

## 6. Targeting the Gut Microbiota–BA Axis for the Treatment of Glycolipid Metabolic Disorders

**Figure 5:**
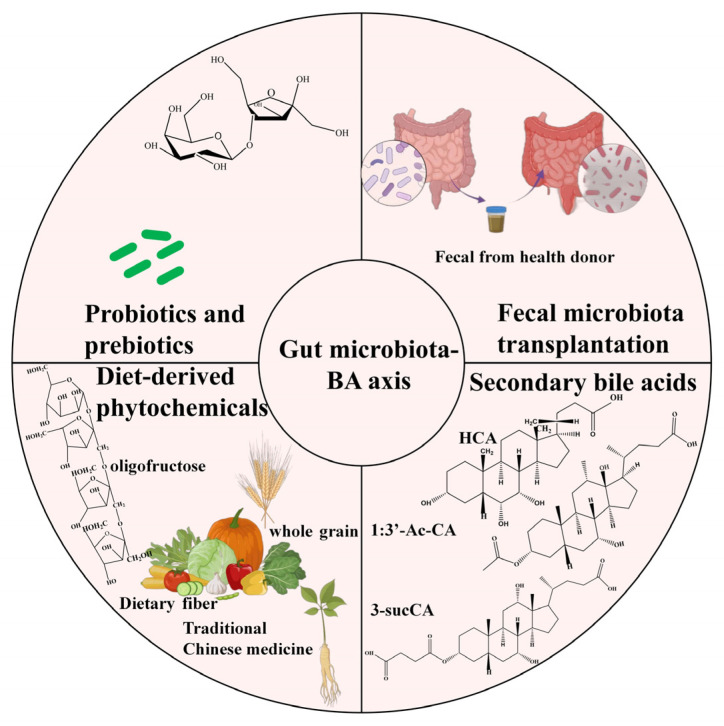
Emerging therapeutic strategies for glucose and lipid metabolism disorders targeting the gut microbiota–BA axis.

Current standard treatments for glucose and lipid metabolism disorders primarily include insulin sensitizers (such as metformin), SGLT-2 inhibitors, GLP-1 receptor agonists, statins, and fibrates. Building upon the critical role of the gut microbiota–BA axis in metabolic regulation as highlighted in this review, targeted modulation of this axis through approaches like regulating BA metabolism, optimizing microbial composition, dietary or probiotic interventions, FMT or activating FXR/TGR5 signaling pathways may offer innovative directions for developing next-generation therapies for glucose and lipid metabolism disorders (Figure 5).

### 6.1. Secondary Bile Acids

BA may provide novel treatment strategies for glucose and lipid metabolism disorders. Research has demonstrated significant depletion of 3-O-acyl-CAs (including 3-acetyl-, 3-propionyl-, 3-butyryl-, and 3-valerylCA) in T2DM patients, indicating their therapeutic potential for T2DM management [1]. In metabolic dysfunction-associated steatohepatitis (MASH) models, 3-succinylCA administration effectively ameliorated disease symptoms through selective enrichment of Akkermansia muciniphila, supporting its development as a targeted molecular therapy [56]. Zheng et al. has established hyocholic acid (HCA) species as both diagnostic biomarkers and multi-target therapeutics for T2DM [79]. These compounds exert dual regulatory effects by activating TGR5 while simultaneously inhibiting FXR signaling, resulting in enhanced GLP-1 secretion and improved glucose homeostasis [80]. Clinical investigations revealed significantly reduced serum levels of specific HCA derivatives (HDCA and glycoHDCA) in metabolic dysfunction-associated fatty liver disease (MAFLD) patients. At the molecular level, HDCA modulates BA metabolism through two distinct mechanisms: first by inhibiting intestinal FXR to stimulate the alternative synthetic pathway, and second by gut microbiota-mediated PPARα activation that suppresses the classical pathway [81]. Further hepatocyte studies identified HDCA’s interaction with ras-related nuclear protein, which upregulates PPARα to promote fatty acid oxidation, ultimately attenuating hepatic inflammation and improving MAFLD pathology [94].

### 6.2. Diet-Derived Phytochemicals

The interplay between diet, genetics, and environmental factors profoundly shapes gut microbiota composition and BA metabolism, with significant implications for metabolic homeostasis. Emerging evidence highlights dietary modification as a promising therapeutic approach, with particular emphasis on fiber supplementation and specific nutritional patterns. The Western diet has been shown to adversely alter BA profiles and promote metabolic dysfunction, including T2DM. In contrast, targeted dietary interventions can beneficially modulate microbial communities and BA metabolism to improve metabolic parameters. Notably, oligofructose supplementation counteracts the Western diet-induced reduction of cecal secondary BAs in murine models while increasing 6α-hydroxylated BA levels through preservation of key bacteria [60]. This soluble fiber acts as a potent prebiotic, undergoing microbial fermentation to generate SCFAs that improve glycemic control in T2DM patients, as evidenced by reductions in fasting glucose, HbA1c, and HOMA-IR [95]. Similarly, whole grain consumption, particularly highland barley (40% inclusion), demonstrates superior metabolic benefits compared to refined grains, attributable to its richer phytochemical content and nutritional completeness [58]. In diabetic mouse models, this intervention significantly lowered fasting blood glucose (FBG) levels, enhanced insulin sensitivity, and fostered the proliferation of beneficial microbes, particularly *Bifidobacterium* and *Akkermansia* (*p* < 0.05) [96]. The ketogenic diet, while effective for weight management, exhibits distinct microbial effects by decreasing BSH-encoding bacteria like *Lactobacillus murinus*. This alteration elevates circulating tauro-conjugated BAs, correlating with improved body weight and glucose regulation [57]. Conversely, high-fat dietary patterns promote dysbiosis and inflammation through BA pool alterations, with specific bacterial species (*Ileibacterium valens* and *Ruminococcus gnavus*) implicated in non-classical BA conjugation pathways affecting intestinal stem cell dynamics [97]. Dietary components significantly influence BA metabolism, as evidenced by studies showing that animal fat consumption increases both BA secretion and fecal secondary BA levels [98,99]. These changes may stimulate glucose-6-phosphate dehydrogenase activity, potentially shifting metabolic flux through the pentose phosphate pathway [100]. Notably, the compound TP mitigates HFD-induced liver steatosis and inflammation by enriching beneficial microbiota, suppressing pathogens. It also enhances BA excretion and transport, reduces BA reabsorption, and stimulates cholesterol efflux, thereby ameliorating HFD-associated hyperlipidemia. Collectively, these results reveal a critical interplay between dietary intake, microbial communities, and metabolic homeostasis, supporting the potential of precision dietary interventions for metabolic disorder management [61].

Traditional Chinese Medicine (TCM) has long recognized the therapeutic value of natural compounds in metabolic regulation, with bear bile representing one of the most historically significant examples. Clinical research has validated UDCA, the active component of bear bile, demonstrating its metabolic benefits, anti-inflammatory properties, and antioxidant effects in T2DM patients through rigorous prospective, double-blind, placebo-controlled trials [101,102]. *Scutellaria baicalensis*, through its flavonoid components, modulates intestinal BA profiles while simultaneously reshaping gut microbiota composition, promoting beneficial bacterial growth while suppressing pathogenic species. This dual action not only optimizes BA metabolism but also fortifies the intestinal barrier and mitigates BA-induced inflammation, potentially offering relief for T2DM-related IR [8]. Forsythia, another important TCM herb traditionally used for its detoxification and anti-inflammatory properties, contains the bioactive compound phillyrin, which demonstrates potential in improving IR [103]. Comparative studies suggest that mature Forsythia may exhibit greater efficacy in detoxification and BA metabolism than its green counterpart, potentially through gut microbiota-mediated regulation of BA pathways [72]. Clinical evidence also supported the efficacy of TCM formulations in diabetes management. For example, the Jiang-Tang-San-Huang pill significantly improved glycemic control and reduced IR in 147 patients while enhancing pancreatic islet function [73]. Its mechanism involves improving gut dysbiosis by enriching BSH-producing bacteria (*Bacteroides*, *Lactobacillus*, and *Bifidobacterium*), leading to ileal accumulation of unconjugated BAs [104]. The Jingangteng capsule demonstrated regulatory effects on BA metabolites and receptors in diabetic models, downregulating lipogenic and pro-inflammatory genes while alleviating hepatic and intestinal inflammation [74]. Tibetan medicine’s Ji-Ni-De-Xie formulation optimized intestinal BA composition and distribution, improving mucosal protection and nutrient absorption [75]. Complementary to herbal interventions, electroacupuncture has shown potential in db/db mice through gut microbiota modulation (elevating Actinobacteria and Firmicutes) and fecal BA pool expansion (notably UDCA and CA), thereby ameliorating metabolic and inflammatory parameters [105]. These findings underscore the critical role of microbiota-driven BA metabolism in IR during T2DM progression, while providing scientific validation for integrating traditional medicine approaches into modern diabetes management strategies.

### 6.3. Probiotics and Prebiotics

Prebiotic and probiotic interventions have emerged as effective strategies for modulating gut microbiota composition to improve glucose and lipid metabolism in metabolic disorders such as obesity and T2DM. Prebiotics are defined as indigestible chemical compounds that selectively nourish beneficial gut bacteria, producing significant health benefits [106]. Plant-derived polysaccharides demonstrate particular promise in diabetes management, with studies showing that daylily polysaccharides effectively lower FBG levels and enhance insulin sensitivity in models of diabetes, while red clover polysaccharides exhibit natural hypoglycemic properties [107]. *Poria cocos* polysaccharides further demonstrate comprehensive metabolic benefits by improving glucose tolerance, reducing inflammation, and strengthening intestinal barrier integrity under HFD conditions. They also alleviated hyperglycemia, hepatic steatosis, and hyperlipidemia through gut microbial regulation [108,109]. Equally noteworthy are mushroom-derived prebiotics, with *Ganoderma lucidum* polysaccharides showing remarkable anti-obesity and anti-inflammatory effects by altering the Firmicutes/Bacteroidetes balance and inhibiting Proteobacteria [110]. Additionally, studies indicate that polysaccharides from *Hirsutella sinensis* alleviate obesity and metabolic dysfunction by selectively promoting the growth of *Parabacteroides goldsteinii* [111]. Polysaccharides derived from *Ganoderma lucidum* spores have shown efficacy in inhibiting obesity, hyperlipidemia, and lipid accumulation in diet-induced obesity (DIO) models through the enrichment of beneficial microbial populations, including *Allobaculum*, Christensenellaceae_R-7_group, and *Bifidobacterium* [112].

Probiotics improve T2DM by enhancing host glucolipid metabolism. *Lactobacillus johnsonii* CCFM1376 effectively ameliorates hypercholesterolemia in mice through modulation of BA metabolism. The treatment group showed significant increases in hepatic–enteric circulation of unconjugated BAs (CA, CDCA, β-MCA, UDCA, and HDCA) and fecal total BA content. Mechanistically, *L. johnsonii* CCFM1376 administration downregulated ileal FXR-FGF15 signaling while upregulating hepatic CYP7A1 expression. These changes, coupled with elevated fecal unconjugated BA levels, confirm that bacterial BSH activity stimulates BA deconjugation while increasing excretion via feces, thereby promoting cholesterol catabolism [63]. Shao et al. demonstrated that *Lactiplantibacillus plantarum* AR113 ameliorates liver steatosis in vitro through a BSH-mediated mechanism. Subsequent investigations revealed that BSH1-knockdown in *L. plantarum* AR113 abolishes its hypoglycemic effects in HFD-fed mice, providing direct evidence for the crucial role of BA metabolism in glucose regulation [113].

Probiotics exert hypoglycemic effects through multiple mechanisms, including modulation of hepatic glucose metabolism and the augmentation of peripheral glucose utilization. *Bifidobacterium animalis* 01 demonstrates significant antidiabetic potential by downregulating key gluconeogenic enzymes (PEPCK and G6Pase) via the IRS/PI3K/Akt pathway in STZ-induced diabetic mice, while simultaneously promoting hepatic glycogen synthesis through GSK-3β and GS regulation [114]. Complementary to this mechanism, *Lactiplantibacillus plantarum* HAC01 improves glycemic control through AMPK/Akt pathway activation, evidenced by increased phosphorylation of these kinases and corresponding suppression of PEPCK/G6Pase expression. Notably, this strain also modulates gut microbiota composition by enriching Akkermansiaceae populations [115]. Additionally, *Bifidobacterium lactis* HY8101 enhances glucose uptake capacity by upregulating GLUT4 expression and insulin sensitivity markers in animal (KK-A(y) mice) models [116].

These studies underscore the therapeutic promise of probiotics and prebiotics in modulating gut microbiota–BA crosstalk for improving glucose and lipid metabolism disorders. However, establishing definitive conclusions regarding their therapeutic efficacy proves challenging owing to substantial heterogeneity and inherent biases in extant clinical investigations. Large-scale, meticulously designed randomized controlled trials are imperative prior to establishing these interventions as standardized therapeutic modalities for metabolic disorders. Notably, the selection of probiotic and prebiotic formulations necessitates personalized approaches, incorporating not only the specific metabolic pathology but also individual variations in BA metabolism profiles and gut microbiota composition.

### 6.4. Fecal Microbiota Transplantation

FMT has shown promise as a clinical strategy that involves transferring healthy gut microbial communities to recipients with dysbiosis, with the potential to not only enhance commensal bacterial function but also fundamentally reconfigure the host microbiome by modifying microbial composition and relative abundance [117]. FMT is now gaining significant traction in metabolic research, with clinical trials actively investigating its efficacy for obesity, T2DM, hyperglycemia, and dyslipidemia [118]. The therapeutic efficacy of FMT is substantiated by its capacity to facilitate successful colonization of beneficial donor-specific microorganisms in the recipient including *Roseburia hominis*, *Blautia lactaris*, and particularly *Akkermansia muciniphila*, with the latter demonstrating a significant correlation with enhanced glucose tolerance. These results suggested that improving gut dysbiosis with FMT may be an effective treatment for obesity. Furthermore, FMT significantly enriches populations of SCFA-producing bacteria such as *Roseburia gutis*, *Bryantella forexigens*, and *Megamonas hypermegale*, which collectively contribute to enhanced insulin sensitivity in patients with metabolic syndrome [119]. Preclinical evidence from Lai et al. discovered that FMT from normal-fat diet donors into HFD-fed mice significantly regulated appetite, reduced body weight, and ameliorated metabolic dysfunction, suggesting its potential to alleviate obesity-related inflammation and metabolic disorders [120]. Research with a highland barley (HB) intervention provides compelling evidence that HB-modulated microbiota exerts significant antidiabetic effects through FMT, as evidenced by profound microbial restructuring (characterized by decreased Firmicutes and increased Bacteroidetes at the phylum level, along with enriched populations of *Bifidobacterium*, *Akkermansia*, *Muribaculum,* and *Duncaniella* at the genus level), successful replication of microbiota profiles from HB-treated mice, and substantial improvements in glycemic control markers, including reduced AUC, GSP, and FBG values following 6-week FMT using microbiota from mice treated with HB for 12 weeks, collectively highlighting FMT’s remarkable capacity to transfer beneficial microbial characteristics and modulate metabolic parameters in metabolic disorders [96].

## 7. Conclusions

Extensive research has been conducted to date investigating the intricate relationship between gut microbiota, microbiota-derived BAs, and glycolipid metabolism. Although numerous reviews have emphasized the BAs and their receptors as key regulators of modulating microbial dynamics, the complex interplay among variations in gut microbiota abundance and functionality, BA levels, newly identified metabolic signaling pathways, and the diverse biological functions of BAs require further systematic exploration. This review systematically highlights the critical importance of gut microbiota in regulating BA metabolism. Through diverse biotransformation reactions, including isomerization, dehydroxylation, and conjugation, gut microbiota substantially expands the structural diversity of BA metabolites. Notably, approximately 40% of secondary BA structures remain chemically uncharacterized. The advent of advanced analytical technologies, particularly high-resolution mass spectrometry and metagenomic sequencing, has revolutionized BA metabolomics research, thereby providing crucial theoretical frameworks and technical platforms for developing next-generation BA receptor-targeted therapeutics. Recent identification of novel BA-based FXR antagonists (e.g., BA-MCY, GUDCA, and TβMCA) has demonstrated their therapeutic potential through gut microbiota remodeling, which differentially modulates intestinal FXR/FGF15 signaling and hepatic FXR activation, ultimately ameliorating metabolic dysregulation. These findings underscore the gut microbiota–BA axis as a promising therapeutic target for metabolic disorders. Future studies should prioritize elucidating the underlying molecular mechanisms and assessing the clinical translatability of these discoveries to facilitate the transition from bench to bedside.

## Figures and Tables

**Table 1:** Summary of treatment strategies for glucose and lipid metabolism disorders via the gut microbiota–bile acid axis.

Treatment Strategy	Intervention	Metabolic Diseases	Mechanism	References
Diet	The ketogenic diet	Obesity	Decreased BSH-encoding bacteria like *Lactobacillus murinus*; elevated circulating tauro-conjugated BAs	[57]
	Whole grain	Whole grain diet	Lowered fasting glucose; enhanced insulin sensitivity; promoted the growth of beneficial microbes including *Bifidobacterium* and *Akkermansia*	[58]
	Buckwheat	T2DM	Increased non-12-OH BA, and decreased 12-OH BAs; activated hepatic FXR/TGR5 signaling	[59]
	Oligofructose	T2DM	Reduced fasting glucose, HbA1c, and HOMA-IR	[60]
	Tomato pectin	HFD-induced hepatic steatosis	Increased TαMCA, TβMCA, TUCDA, and TCDCA levels; inhibited the intestinal FXR/FGF15 pathway; activated hepatic FXR	[61]
	Capsaicin	T2DM	Suppressed BSH activity and reduced the abundance of *Lactobacillus*; elevated TβMCA levels; inhibited enterohepatic FXR/FGF15 pathway and subsequently expanded the BA pool through upregulated CYP7A1 expression and enhanced hepatic BA synthesis	[62]
Probiotics	*Lactobacillus johnsonii* CCFM1376	Hypercholesterolemia	Inhibited ileal FXR-FGF15 signaling and upregulated hepatic CYP7A1 expression	[63]
	*Lactiplantibacillus plantarum 104*	High-fat-diet-induced dyslipidemia	Increased the abundance of *Bacteroides*, *Akkermansia*, *Lactobacillus*, and *Clostridium* and decreased the abundance of *Oscillospira* and *Coprococcus*; increased the ileal TαMCA, TβMCA and TUDCA; inhibited ileal FXR-FGF15 pathway	[64]
	*L*. *plantarum* H-87	High-fat induced obesity	Increased BSH bacteria; hydrolyzed GCDCA and TUDCA, inhibited TGR5 signaling and GLP-1 secretion; suppressed insulin hypersecretion and alleviated IR	[65]
	*Christensenella minuta*	High-fat-diet	*Gut commensal Christensenella minuta* generated 3-O-acylated secondary BAs; inhibited intestinal FXR activity	[1]
Plant-derived polysaccharides	Mulberry leaf polysaccharides	T2DM	Enhanced the abundance of *Prevotella*, *Ruminococcus*, and *Lactobacillus*; enhanced mRNA expression of Cyp7a1 and Cyp8b1, and ileal TGR5; suppressed hepatic and ileal FXR	[66]
	Platycodonis radix polysaccharides	Obesity	Enhanced the relative abundances of bacteria involved in the production of secondary BAs, such as *Lachnospiraceae_NK4A136* and *Eubacterium coprostanoligenes*; inhibited ileal FXR-FGF15 signaling	[67]
mushroom-derived polysaccharides	Polysaccharide NAP-3	T2DM	Increased *Akkermansia abundance*; suppressed BSH activity and reduced the abundance of *Lactobacillus*; elevated TβMCA levels; inhibited FXR and activated TGR5; induced the release of GLP-1	[68]
The polysaccharide from *Lyophyllum decastes*		Obesity	Modulated the gut microbiota, increased HDCA, DCA, and LCA levels; activated TGR5; promoted BAT thermogenesis and sWAT browning; enhanced energy expenditure	[59]
Traditional Chinese Medicine	Gyejibongnyeong-hwan	Western diet-induced dyslipidemia	Modulated the gut microbiota composition; reduced CDCA and LCA levels; inhibited intestinal FXR-FGF15 signaling and upregulated hepatic genes involved in cholesterol metabolism (LXRα, ABCG8) and BA synthesis (CYP7A1)	[69]
	Salidroside	Metabolic dysfunction-associated steatotic liver disease (MASLD)	Modulated the gut microbiota composition; lowered TαMCA and TβMCA levels and elevated βCDCA levels; activated FXR	[70]
	*Scutellaria baicalensis*	T2DM	Modulated the gut microbiota composition; altered intestinal BA profiles	[8]
	dicaffeoylquinic acids	T2DM	Increased bacteria with BSH activity (e.g., *Acetatifactor sp*011959105 and *Acetatifactor muris*); increased the content of TβMCA; inhibited intestinal FXR-FGF15 signaling	[71]
	*Forsythia suspensa*	T2DM	Regulated gut microbiota and BAs metabolism; improved insulin resistance	[72]
	Jiang-Tang-San-Huang pill	T2DM	Enriched BSH-producing bacteria (*Bacteroides*, *Lactobacillus*, *Bifidobacterium*), and increased unconjugated BAs	[73]
	Jingangteng	T2DM	Regulated gut microbiota composition and BA metabolites; downregulated lipogenic and pro-inflammatory genes; alleviated hepatic and intestinal inflammation	[74]
	Ji-Ni-De-Xie formulation	T2DM	Modulated the gut microbiota and increased CA and UDCA levels; reduced inflammation	[75]
	Zhi-Kang-Yin formula	High-fat diet-induced metabolic disorders	Increased BSH-producing bacteria (e.g., *Bifidobacterium*) and elevated unconjugated BAs	[76]
	QiDiTangShen	Diabetic nephropathy	Reduced the abundance of Lachnospiraceae_NK4A136_group, *Lactobacillus*, and *Bacteroides* and increased the abundance of *Alloprevotella*; lowered β-MCA, TCA, Tβ-MCA, and DCA levels	[77]
	Simiao Wan	HFD-induced hyperlipidemia	Suppressed BSH-producing bacteria; elevated T-β-MCA and TUDCA levels; inhibited ileal FXR-FGF15 pathway and activated the hepatic CYP7A1/FXR/SHP axis; promoted BA efflux	[78]
Bile acids	3-succinylCA	MASH	Modulated the gut microbiota composition by increasing the abundance of *Akkermansia muciniphila*	[56]
	HCA	T2DM	HCA was formed through modification by the gut microbiota; activated TGR5 and inhibited FXR signaling; enhanced GLP-1 secretion and improved glucose homeostasis	[79,80]
	HDCA	Metabolic dysfunction-associated fatty liver disease	Stimulated hepatic alternative BA synthetic pathway by inhibiting intestinal FXR, and suppressed the hepatic classical BA synthetic pathway by modulation of gut microbiota to activate PPARα signaling pathway	[81]
	3-O-acylated secondary BAs	Obesity	*Christensenella minuta* generated 3-O-acylated secondary BAs; inhibited intestinal FXR	[1]
	BA–methylcysteamine	Hypercholesterolemic	Modulated the gut microbiota to promote BA–MCY production; inhibited FXR; elevated BA production; reduced hepatic lipid accumulation	[82]
	GUDCA	Obesity	Increased the abundance of *Bacteroides vulgatus*; Increased TLCA levels and activated TGR5; induced GLP-1 secretion; enhanced energy expenditure	[27]
	GUDCA	High-cholesterol-fed ApoE−/− mice	Increased the abundance of *Alloprevotella* and *Parabacteroides*; inhibited the intestinal FXR signaling, reduced blood ceramide levels	[83]
